# Host‐associated genetic differentiation and origin of a recent host shift in the generalist parasitic weed *Phelipanche ramosa*

**DOI:** 10.1002/ece3.10529

**Published:** 2023-09-11

**Authors:** Valérie Le Corre, Carole Reibel, Vaya Kati, Stéphanie Gibot‐Leclerc

**Affiliations:** ^1^ Agroécologie, INRAE, Institut Agro, Univ. Bourgogne Franche‐Comté Dijon France; ^2^ Faculty of Agriculture, Forestry and Natural Environment Aristotle University of Thessaloniki Thessaloniki Greece

**Keywords:** approximate Bayesian computation, broomrape, host shift, host‐associated genetic differentiation, microsatellites, phylogeny

## Abstract

Branched broomrape, *Phelipanche ramosa* (L.) Pomel, is a globally distributed parasitic weed of economic importance. In Europe, where it is native, it can infest several crops, notably tomato, tobacco, and hemp. In western France, it has recently adapted to a new host crop, oilseed rape, causing substantial damage. The aim of this study was to investigate the evolutionary relationships and genetic differentiation among *P*. *ramosa* populations infesting different hosts. We collected 1611 *P*. *ramosa* samples from 109 fields cultivated with six different crops (oilseed rape, tobacco, hemp, tomato, lentil, and celery) and distributed among six European countries. All samples were genotyped for ten microsatellite loci and a subset of samples was sequenced for two nuclear genes and two chloroplast genes. Genetic differentiation among populations was high (*F*_ST_ = 0.807) and mainly driven by differentiation among different host crops, with no significant geographic structure. Genetic structure analysis identified up to seven biologically meaningful clusters that matched with host crops of origin. Reconstructed networks of sequence haplotypes and multilocus SSR genotypes showed a large genetic divergence between samples collected on oilseed rape and samples collected on other crops. The phylogeny inferred from DNA sequences placed samples collected from oilseed rape as a basal lineage. Approximate Bayesian Computations were used to compare different evolutionary scenarios of divergence among the three main genetic clusters, associated, respectively, with oilseed rape, tobacco, and hemp as host crops. The best‐supported scenario indicated that *P*. *ramosa* infesting oilseed rape derived recently from an ancient, unknown lineage. Our results suggest that a more complete description of the genetic diversity of *P*. *ramosa* is still needed to uncover the likely source of the recent adaptation to oilseed rape and to anticipate future new host shifts.

## Graphical abstract

Branched broomrape, *Phelipanche ramosa*, is a parasitic weed that can infest several crops, notably tobacco, hemp, and tomato. In western France, it has recently adapted to a new host, oilseed rape. We describe the genetic diversity of populations sampled from six different crops across Europe. We observed strong host‐associated genetic differentiation and found that the host shift toward oilseed rape involved a recent divergence from an ancient, unknown lineage.

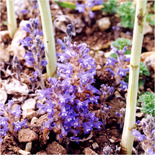

## 1 INTRODUCTION

**FIGURE 1:**
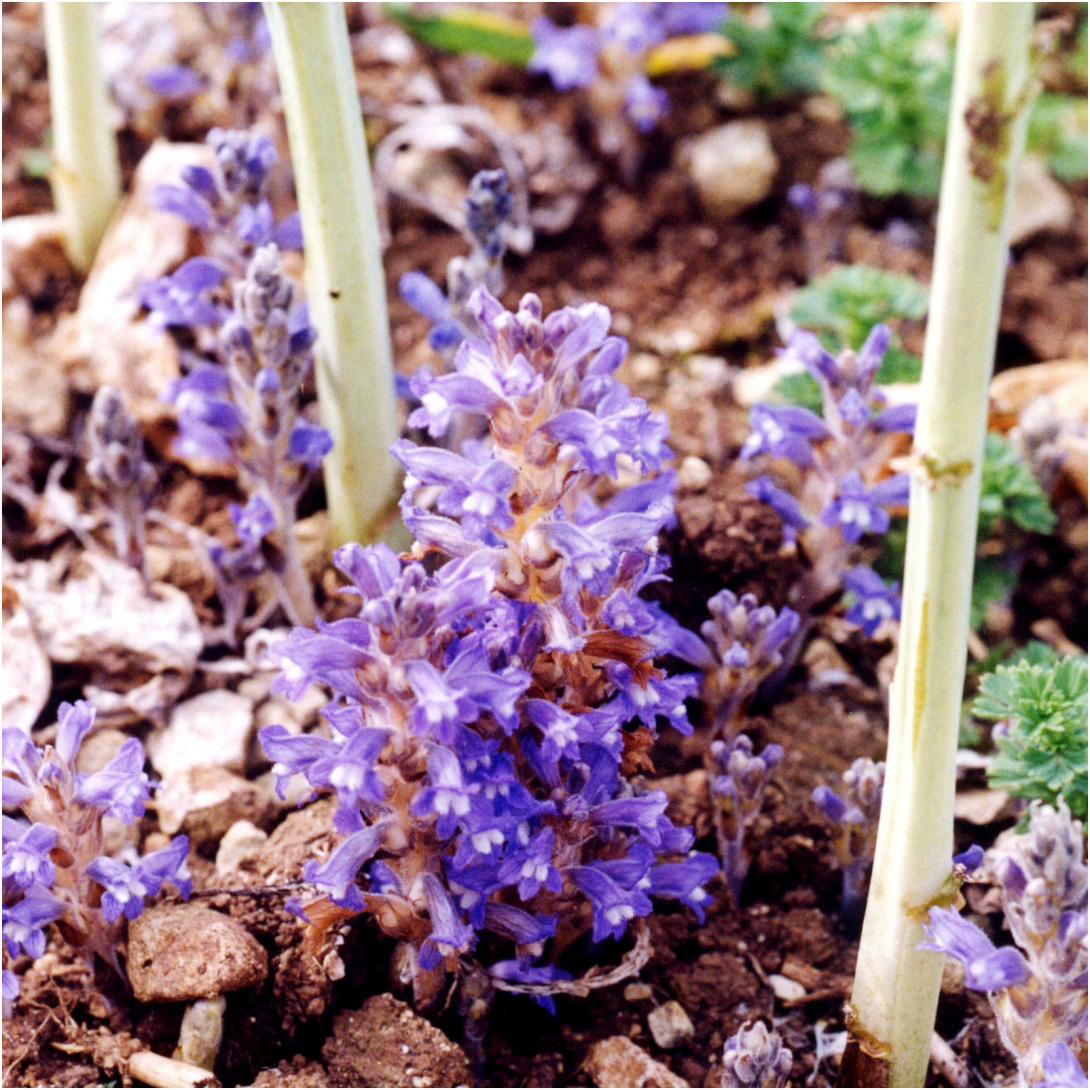
*Phelipanche ramosa* flowering in an oilseed rape field.

Parasitic flowering plants have evolved several times and represent 1%–2% of all angiosperms (Nickrent, 2020). They are characterized by a specialized organ called the haustorium, which allows direct connection to the vascular system of a host plant and the ability to extract nutrient resources from the host. Among parasitic plants, holoparasites have no photosynthetic activity and are entirely dependent on their hosts for nutrition. The *Orobanchae* is a tribe within the *Orobanchaceae* family that contains only holoparasites, among which the broomrapes (genera *Phelipanche* and *Orobanche*) have been much studied because they include root parasites that attack broad‐leaved crops and can cause severe yield losses. These agronomically important parasitic plants vary widely in their host range. Some are highly specialized, while others have a broad, diversified host range (Hu et al., 2020). *Phelipanche ramosa* (L.) Pomel, the branched broomrape, is the most widespread broomrape in the world. It originates from Europe and Asia and was introduced in Australia, China, North America, and South America (Parker, 2009). It is a noxious parasitic weed that can infest over 50 host species, including many crops, such as tobacco, tomato, hemp, and some leguminous species, particularly in countries surrounding the Mediterranean basin and in Central Europe (Parker, 2013). The host range of *P*. *ramosa* includes also several weed species (Qasem & Foy, 2007). In western France, a massive expansion of *P*. *ramosa* to oilseed rape fields has been observed since the beginning of the 1990s (Brault et al., 2007; Gibot‐Leclerc et al., 2003) causing heavy yield losses (Figure 1). More recently, infestation of oilseed rape fields was also reported in northern Greece (Tsialtas & Eleftherohinos, 2011).

In two previously published studies, microsatellite markers were used to investigate patterns of genetic differentiation among agricultural populations of *P*. *ramosa* in relation to the host crop of origin (Le Corre et al., 2014) and in relation to the results of cross‐infestation assays with different host crops (Stojanova et al., 2019). Both studies reported a high level of host‐associated genetic differentiation. Remarkably, *P*. *ramosa* accessions collected in western France and capable of infecting oilseed rape formed a highly divergent group with low genetic diversity. Two other less distinct, more genetically diverse groups were observed, that preferentially infected hemp and tobacco, respectively. The restricted spatial distribution and recent nature of infestations on oilseed rape suggest a recent shift in host preference. However, this scenario is at odds with the high genetic divergence observed between the “oilseed rape” genetic group and other genetic groups. In the present study, we re‐examine the patterns of genetic differentiation of *P*. *ramosa* in relation to its host crops using a large set of European populations collected from six different crops. We combine phylogenetic inference based on DNA sequences, population genetic structure based on microsatellite marker data, and evolutionary scenario testing using Approximate Bayesian Computations to elucidate the evolution of host specialization in branched broomrape.

## 2 MATERIALS AND METHODS

### 2.1 Population sampling and DNA extraction

**FIGURE 2:**
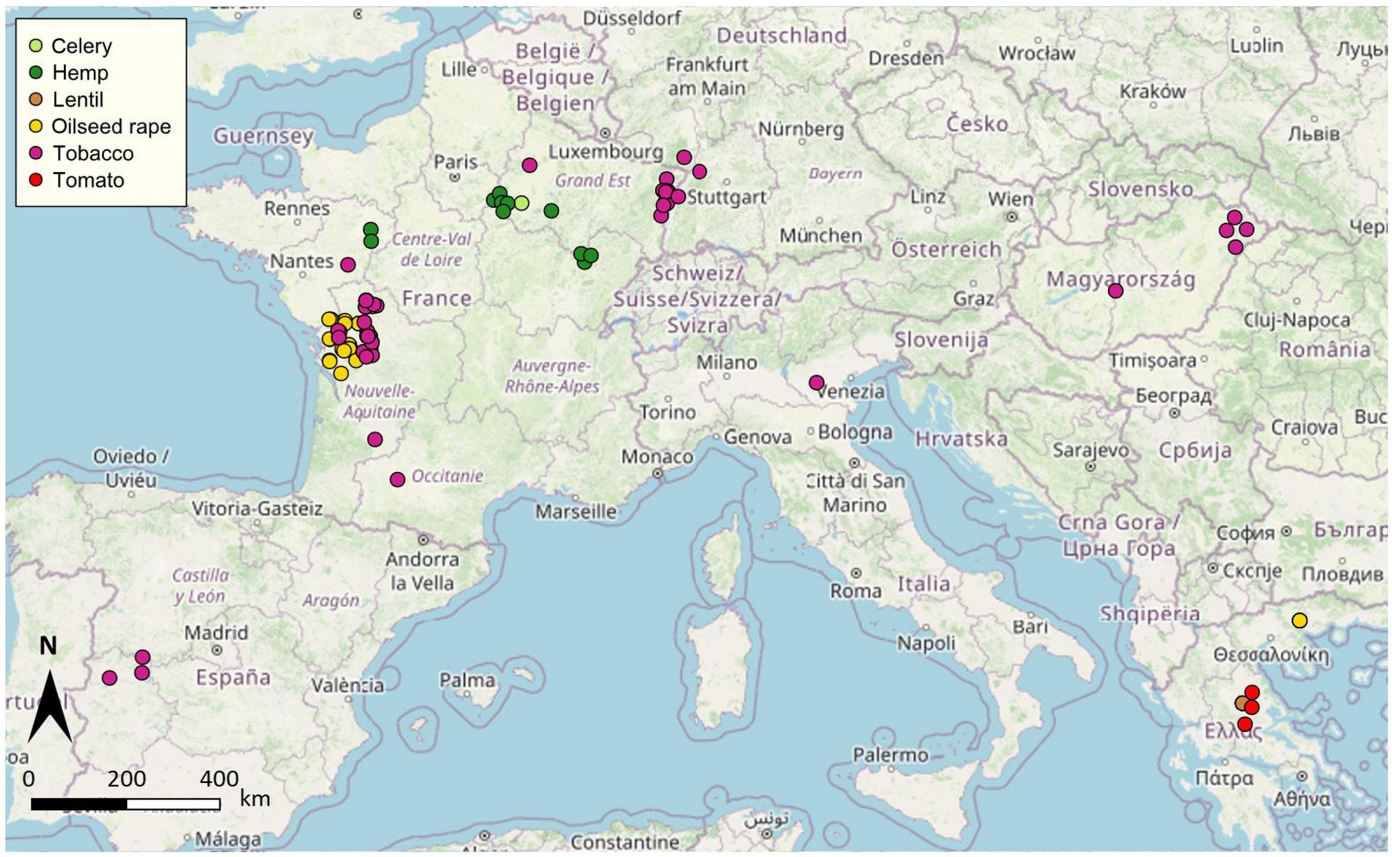
Map showing the geographical locations and associated host crops for the 109 populations of *Phelipanche ramosa* sampled for this study.

*Phelipanche ramosa* plants were collected within 109 cultivated fields across Europe, from 1999 to 2018. Samples were collected as emerged *P*. *ramosa* stems that were spaced at least one meter apart, to ensure that only one individual was collected from each host plant. The samples were individually coded and stored in paper bags at −20°C until further analysis. The aim was to collect at least 10 plants per field. In the end, the number of plants per field varied from 1 to 48, with a median of 11. In the following, we will use the term “population” to refer to the set of *P*. *ramosa* plants collected from the same agricultural field and from a single host crop. Populations were distributed among 6 countries (France, Spain, Italy, Germany, Hungary, Greece) and were sampled in six different host crops (Figure 2): tobacco (60 populations), oilseed rape (25 populations from western France and 4 populations from Greece), hemp (11 populations), lentil (5 populations), processing tomato, hereafter tomato (3 populations), celery (1 population). A small fragment of the stem of each individual plant collected was used for DNA extraction. The total genomic DNA was extracted using a rapid method based on incubation at high temperature in a Tris–HCl‐EDTA buffer, as described in Délye et al. (2009).

### 2.2 DNA sequencing and microsatellite genotyping

A set of 47 plants, each from a separate population and representing all the host species and geographical regions sampled were selected for DNA sequencing. Two chloroplast regions, the rpl16 intron and the trnK‐trnQ intergenic region were sequenced using custom primers (rpl16F: AGTCTATCTGAAAACCAACCCG, rpl16R: TCTCAGTTACTCCGCCCATTTC and trnK‐trnQF: GGCAGAACTAAATAGACCACGTTC, trnK‐trnQR: TAGGGAAGGGTGTTGAGTTAATTC) designed based on the available *P*. *ramosa* complete chloroplast genome sequence (GenBank accession HG803180.1). The internal transcribed spacer (ITS) of nuclear ribosomal DNA was PCR‐amplified following Schneeweiss et al. (2004). Obtained sequences contained part of the small subunit ribosomal DNA ITS1, the 5.8 s ribosomal gene, ITS2, and part of the large subunit ribosomal DNA. We sequenced a second nuclear gene, the B01 transposase gene, a member of the hAT‐like transposon superfamily identified by Sun et al. (2016), using their primer pairs OhAT‐AF2/OhAT‐AR. Sanger sequencing of purified PCR products was performed by Genewiz Germany GmBH. Microsatellite genotyping data were obtained for the 109 sampled populations and 1611 individuals (from 1 to 48 individuals per population, mean 14.8). Ten microsatellite markers described in Le Corre et al. (2014) were used: SSR_phera02, SSR_phera14, SSR_phera18, SSR_phera19, SSR_phera20, SSR_phera28, SSR_phera40, SSR_phera46, SSR_phera46 and SSR_phera53. Total genomic DNA extraction, PCR amplifications, and capillary electrophoresis were performed as described in Le Corre et al., 2014. Fragment size scoring was performed with the software Peak Scanner 1.0 (Applied Biosystems).

### 2.3 Network and phylogenetic analyses on gene sequences

The sequences for the two chloroplast loci and the two nuclear loci were trimmed in MEGA version 11 (Tamura et al., 2021) and aligned using ClustalW in MEGA 11. A haplotype network was constructed separately for each locus using the median‐joining network method implemented in PopArt v1.7 (Leigh & Bryant, 2015). *Phelipanche aegyptiaca* was used as an outgroup for the phylogenetic analyses. For the *rpl16* intron and *trnK‐trnQ* chloroplast regions, homologous sequences were retrieved from the available chloroplast complete genome sequence (Genbank accessions KU212370.1). For the *ITS* sequences, we used GenBank accession MG948171.1. For *BO1*, we used GenBank accession KM037755.1. Due to low levels of polymorphism detected (see Section 3), data from *rpl16*‐intron was discarded from phylogenetic analyses. A concatenated data set containing the unique haplotypes defined by the *trnK‐trnQ*, *ITS*, and *BO1* sequences was used to infer a Bayesian phylogeny using MrBayes v3.2.7 (Ronquist et al., 2012). A partitioned analysis was conducted using the 2‐parameters Kimura substitution model with a gamma distribution of evolutionary rates (K2 + G) and with separate estimations of parameters for each of the 3 loci. We used default priors and performed two independent runs of 500,000 Markov chain Monte Carlo generations. The first 25% generations were discarded as burn‐in and diagnostics were computed every 1000th generation. Convergence and stationarity were checked as recommended (Nascimento et al., 2017), based on the standard deviation of split frequencies, on effective sample size, and potential scale reduction factor for the estimated parameters. The tree was visualized, midpoint‐rooted, and edited using FigTree v1.4.4 (http://tree.bio.ed.ac.uk/software/figtree/).

### 2.4 Genetic diversity and population structure

Genetic diversity statistics based on microsatellite data were estimated for each of the 75 populations within which at least ten individuals were genotyped. The mean and effective number of alleles and the observed and unbiased expected heterozygosities were estimated using GENALEX v6.5 (Peakall & Smouse, 2006, 2012). Multilocus genotypes (MLG) were identified using the R package poppr v2.9.2 (Kamvar et al., 2015) after filtering out genotypes with any missing data. To account for genotyping errors, unique multilocus genotypes were collapsed using Bruvo‘s genetic distance and the conservative farthest neighbor algorithm. Observed and expected number of MLGs as well as Simpson's diversity index where estimated within each population with at least 8 genotypes without any missing data. Observed and expected number of MLGs (at a common sample size of 50) were also calculated separately for each set composed of the individuals associated with each host crop. The R package hierfstat (Goudet, 2004) was used to estimate Weir and Cockerham fixation's indices *F*_ST_ and *F*_IS_ (Weir & Cockerham, 1984) and their 95% bootstrap confidence intervals (999 bootstraps). The effects of the host crop and geography on the genetic differentiation among populations were investigated more in details for the three most extensively sampled host crops: hemp, oilseed rape from France and tobacco. An analysis of molecular variance (AMOVA) was performed with the three main host crops and populations within host crops as nested hierarchical levels, using the R package hierfstat (Goudet, 2004). In addition, to compare the levels of differentiation among populations at the global level and within each host crop, we computed Jost's *D* (Jost, 2008) values and their 95% confidence intervals (999 bootstraps) using the R package mmod. Jost's *D* is better suited than *F*_ST_ for comparing levels of differentiation among datasets with different numbers of populations (Alcala & Rosenberg, 2019). Mantel's tests were performed separately for each host crop to evaluate the correlation between pairwise Weir and Cockerham's *F*_ST_ values and great‐circle pairwise geographic distances among sampled fields, using the R package ade4 with 10^3^ random permutations to assess significance.

### 2.5 Genetic clustering and migration

The presence of genetic clusters was assessed without considering the stratification of samples into cultivated fields and host crops, using a discriminant analysis of principal components (DAPC) with the package adegenet (Jombart et al., 2010). The DAPC method does not assume unlinked markers nor Hardy–Weinberg equilibrium and is thus well suited for a predominantly inbreeding species. The optimal number of genetic clusters was searched based on the Bayesian Information Criterion (BIC). As continuously decreasing BIC values were observed, we investigated the successive patterns revealed by considering from 2 to 7 clusters. Considering more than 7 clusters led to more complex, biologically less meaningful patterns. As recommended (Jombart et al., 2010), the a‐score was used as a criterion for choosing the optimal number of principal components to keep in each analysis.

First‐generation migrants were detected in GeneClass v.2.0 (Piry et al., 2004), based on the data set for the 75 populations with at least 10 individuals genotyped and no missing data. As recommended when not all source populations have been sampled, we used the test statistic *L*_H_, which is the likelihood of observing a genotype in the population in which it was sampled (Paetkau et al., 2004). We selected the Rannala and Mountain (1997) criterion and the resampling method of Paetkau et al. (2004) to determine the critical value of *L*_H_ based on 10,000 simulated genotypes. Only individuals with a probability <.01 were considered as putative migrants.

### 2.6 Comparison of evolutionary scenarios using approximate Bayesian computations (ABC)

Hypothetical evolutionary scenarios for the genetic clusters detected in *P*. *ramosa* were compared using an ABC approach in DIYABC Random Forest v1.0 (Collin et al., 2021). In the first step, we investigated the six possible scenarios involving the three main genetic clusters detected with DAPC: OR (oilseed rape), TB (tobacco) and HP (hemp), and two divergence events among them (Appendix B: Figure A5). Priors were as follows: all effective sizes were drawn from a uniform distribution with bounds of 10 and 1000; the most ancient divergence event was drawn in a Uniform distribution bounded by 100 and 100,000 generations; the most recent divergence event was drawn in a Uniform distribution bounded by 10 and 10,000 generations. The training set included a total of 60,000 simulated data sets, that is, 10,000 per scenario. Each data set was summarized using 39 summary statistics as well as the first three most discriminant linear combinations of these summary statistics. A Random Forest tree‐based classification method was used to identify the best scenario and estimate its posterior probability, as well as the global and local error rates. Ten replicate sets of 1000 trees per scenario were generated and the scenario that received the highest number of votes was selected. To infer demographic and historical parameters, we considered the best scenario with a slight modification: a ghost (unsampled) population was introduced, from which group OR (Oilseed Rape) was considered to have diverged (Appendix B: Figure A7). Prior distributions of the parameters were Uniform with their bounds chosen so that observed values of the summary statistics fell within the simulated values, using the “Prior and scenario checking” option in DYABC‐RF and 2000 simulated data. Priors for the effective sizes of the 3 genetic groups OR, TB, and HP, and the ghost population were bounded by 10 and 1000. Priors for the most ancient divergence event, that between the ghost population and HP, were bounded by 1000 and 50,000 generations; priors for the divergence event between HP and TB were bounded by 10 and 5000 and priors for the divergence event between the ghost population and OR by 10 and 1000. A total number of 50,000 data sets was simulated to generate the posterior distributions of the parameters. For each parameter, we computed the median as point estimate and a 95% confidence interval. Global and local accuracy indices corresponding to global and local Normalized Mean Absolute Error (NMAE, Collin et al., 2021) were estimated using out‐of‐bag estimators from a random sample of 10,000 data. The number of trees in the constructed random forest was fixed to 1000, as this number turned out to be large enough to ensure a stable estimation of the global accuracy metrics. For each parameter, we conducted ten replicate RF analyses based on the same training set.

## 3 RESULTS

### 3.1 Sequence divergence and phylogenetic inference

**FIGURE 3:**
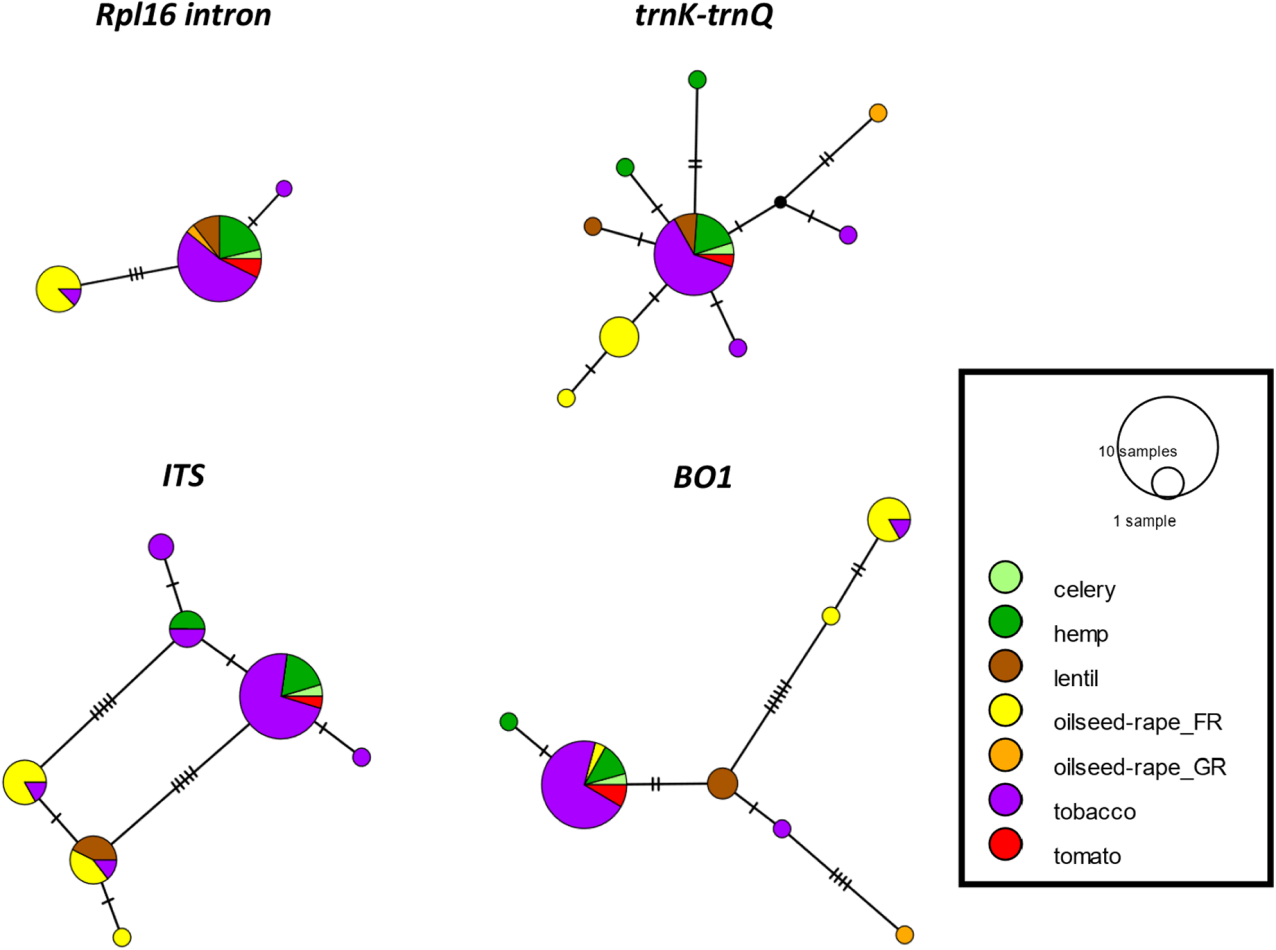
Median‐joining networks inferred from DNA sequences at two chloroplast loci (*rpl16* intron and *trnK‐trnQ*) and two nuclear loci (*ITS* and *BO1*). Haplotypes are colored according to the host crop from with the samples were collected.

After elimination of incomplete or low‐quality chromatograms, the sequence data consisted of 37 sequences for *rpl16*‐intron, 36 sequences for *trnK‐trnQ* intergenic spacer, 44 sequences for *ITS*, and 40 sequences for *BO1*. The chloroplast *rpl16*‐intron had sequence lengths of 898 bp with 4 (0.44%) variable sites. The chloroplast *trnK‐trnQ* marker had sequence lengths of 1007 bp with 11 (1.1%) variable sites. The *ITS* sequences had a length of 842 bp with 9 (1%) variable sites. The *BO1* sequences had a length of 1018 bp with 14 (1.37%) variable sites. Only three haplotypes were identified among the rpl16‐intron sequences, while 9 haplotypes were identified among the trnK‐trnQ sequences and 7 haplotypes were identified among each of the nuclear sequences (Figure 3). For each locus, a major common haplotype was observed, grouping samples collected on different host crops (tobacco, hemp, celery, and tomato, and sometimes other crops depending on the dataset) and from different geographical origins (France, Germany, Greece, and Hungary, and sometimes Spain and Italy depending on the dataset). The samples collected on oilseed rape in France were mostly grouped into one or two common haplotypes showing a clear divergence from the main haplotype.

**FIGURE 4:**
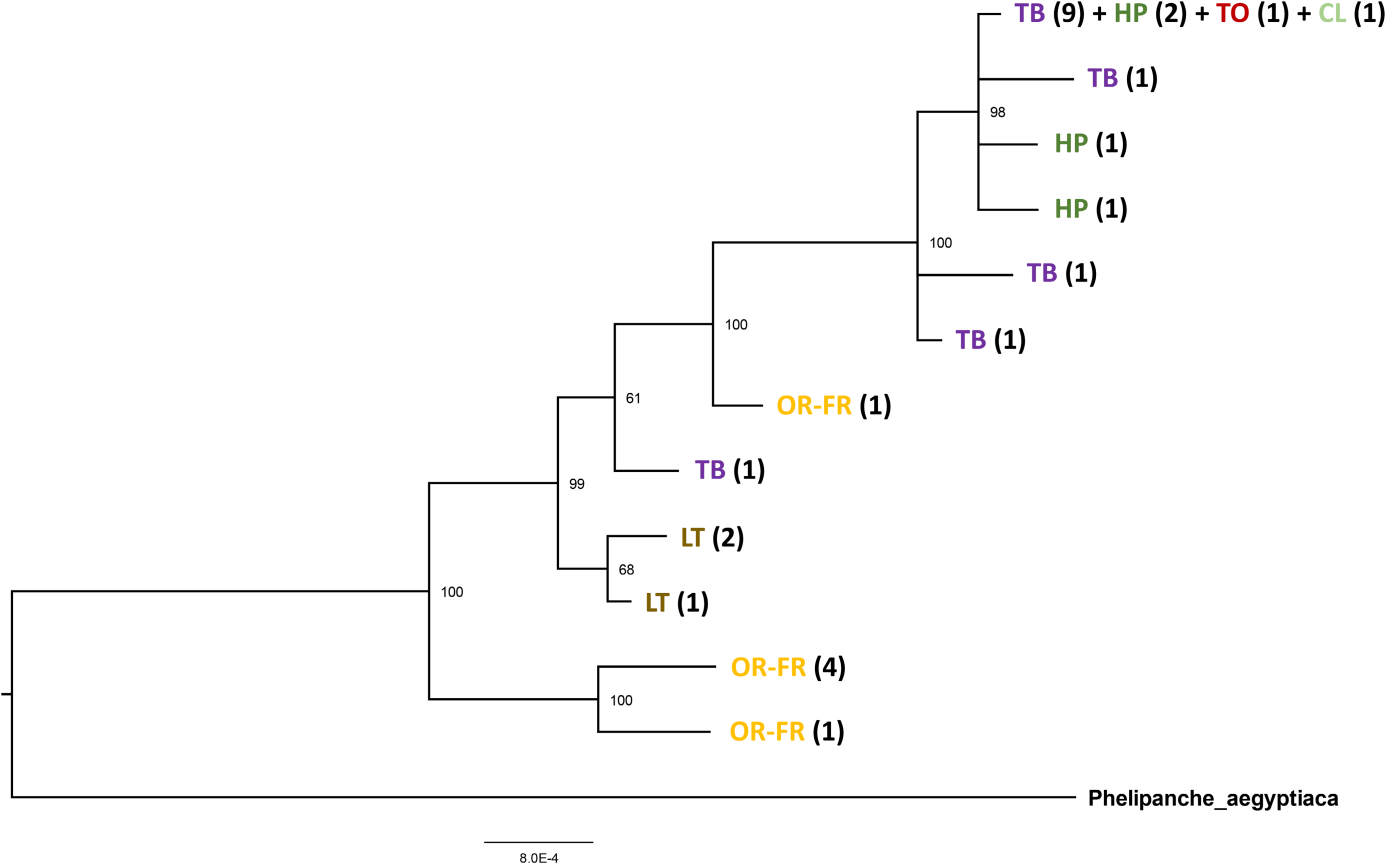
Midpoint‐rooted Bayesian inferred tree for the 12 *Phelipanche ramosa* haplotypes obtained among 28 individuals sequenced for *trnK‐trnQ*, *ITS*, and *BO1*. Branch lengths are proportional to the inferred divergence levels. Bayesian posterior probabilities (percent) are indicated at each node. Host crops from which the haplotypes were sampled are indicated as follows: OR‐FR, oilseed rape from France; LT, lentil; TB, tobacco; HP, hemp; TO, tomato; CL, celery. The numbers within parentheses indicate the number of individuals having that haplotype.

Sequences at the three most informative loci (*trnK‐trnQ*, *ITS*, and *BO1*) were concatenated for their 28 common samples and a Bayesian phylogeny was inferred for the 12 haplotypes identified among them (Figure 4). The phylogenetic tree showed several well‐supported lineages (posterior probability >.95). The basal lineage contained all the samples collected from oilseed rape fields in France, except for one sample, phera41, which formed a separate branch at an intermediate position in the tree. A second lineage grouped samples collected from lentil fields in Greece. A third lineage clustered 18 of the 28 samples sequenced. All host crops except oilseed rape and lentil, and various countries of origins (France, Germany, Greece, Hungary) were represented in this third lineage.

### 3.2 Genetic variation within and among populations and host crops

Each of the ten microsatellite markers detected from 3 alleles to 22 alleles among the 1611 individuals genotyped (mean across loci: 8.3). Observed heterozygosity was very low at all markers (from 0 to 0.03, mean 0.013), as expected under a predominantly selfing mating system. The estimated value of the fixation index *F*is was 0.887 (95% CI: 0.5702–0.9855). Low levels of genetic diversity were detected within most populations (Table 1). The effective number of alleles per population varied between 1.0 and 2.7 (mean 1.2), the unbiased expected heterozygosity varied between 0.008 and 0.569 (mean 0.109).

**TABLE 1:** Within‐population allelic diversity and genotypic diversity, summarized for each host crop separately.

	Celery	Hemp	Lentil	Oilseed rape FR	Oilseed rape GR	Tobacco	Tomato
Allelic diversity							
No. of populations	1	9	3	21	4	33	3
Na	1.10	1.34 (0.022)	1.47 (0.038)	1.47 (0.015)	3.10 (0.104)	1.44 (0.010)	1.7 (0.067)
Ne	1.01	1.06 (0.004)	1.16 (0.046)	1.14 (0.005)	2.13 (0.092)	1.22 (0.007)	1.32 (0.056)
He	0.01	0.04 (0.002)	0.09 (0.015)	0.09 (0.003)	0.41 (0.029)	0.10 (0.003)	0.16 (0.030)
Genotypic diversity							
No. of populations	1	8	3	21	4	15	2
MLG	2	3.89 (0.211)	6.00 (0.882)	4.00 (0.181)	10.00 (0.957)	3.40 (0.133)	7.50 (1.061)
eMLG	1.42	2.53 (0.071)	3.92 (0.460)	2.80 (0.071)	6.08 (0.351)	2.80 (0.105)	3.87 (0.122)
Lambda	0.10	0.41 (0.015)	0.61 (0.077)	0.40 (0.013)	0.82 (0.028)	0.43 (0.017)	0.65 (0.034)

Abbreviations: eMLG, expected number of multilocus genotypes; He, average unbiased expected diversity; Lambda, Simpson's diversity; MLG, number of multilocus genotypes; Na, number of alleles per locus; Ne, effective number of alleles per locus.

**FIGURE 5:**
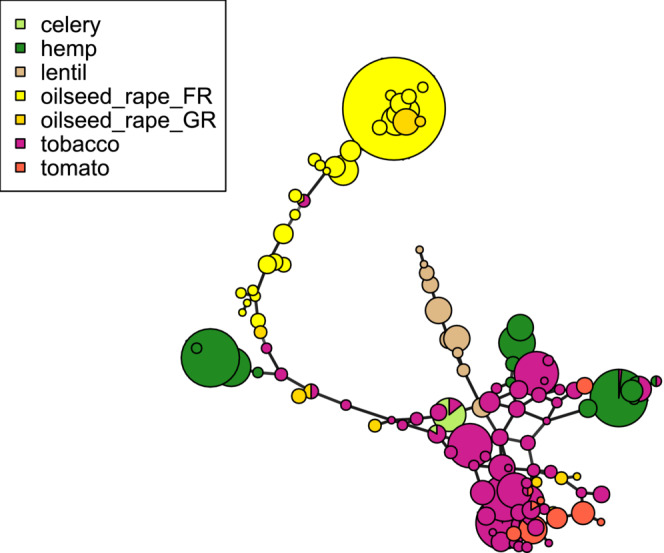
Minimum spanning network of microsatellite genotype data based on Bruvo's distance. Each node represents a unique, non‐singleton multilocus genotype. Node sizes are a function of the number of individuals carrying the genotype. Node colors represent the host crop from which individuals were sampled. Oilseed_rape_FR: oilseed rape fields from France; oilseed_rape_GR: oilseed rape fields from Greece.

A total of 194 multilocus genotypes were observed across the whole data set. Most MLGs were rare, as 102 were singletons (observed in only one individual), and only three were observed at a frequency higher than 5%. The most frequent MLG had an overall frequency of 19.7% and was observed in samples collected from oilseed rape in France, representing 56% of them. Among the 92 non‐singleton MLGs, only 14 (15%) were shared between populations collected on different host crops. All the shared MLGs were observed in both tobacco and a single other crop, which could be tomato, hemp, celery, oilseed rape from Greece or oilseed rape from France (Table 2). The expected number of MLGs based on a rarefaction analysis to a sample size of 50 (Table 2) was higher for plants sampled from tobacco fields (eMLG = 23.1) than for plants sampled from French oilseed rape fields (eMLG = 13.6) or hemp fields (eMLG = 11.7). At the within‐population level, genotypic diversity was markedly higher for the four populations collected in oilseed rape fields in Greece, but similar among the three main host crops, hemp, oilseed rape from France and tobacco (Table 1). A minimum spanning network of all non‐singletons MLGs is shown in Figure 5. The genotypes collected in oilseed rape fields in France formed a divergent lineage, with the most frequent MLG being the most divergent. Most samples collected on tobacco, tomato, hemp, and lentil formed a reticulated cluster. Genotypes collected in oilseed rape fields in Greece were distributed across the entire network.

**TABLE 2:** Number of multilocus genotypes (MLGs) observed at ten microsatellite markers among individuals sampled from different host crops.

	Celery	Hemp	Lentil	Oilseed rape FR	Oilseed rape GR	Tobacco	Tomato
*N*	19	236	64	353	65	412	50
Shared	2 (100%)	4 (17%)	0 (0%)	1 (2%)	2 (5%)	14 (20%)	5 (42%)
Host‐specific	0	9 (39%)	7 (43%)	22 (48%)	8 (21%)	30 (42%)	0 (0%)
Singletons	0	10 (43%)	9 (56%)	23 (50%)	28 (74%)	27 (38%)	7 (58%)
Total MLGs	2	23	16	46	38	71	12
eMLG	–	11.7	14	13.6	31.1	23.1	12

Abbreviations: eMLG, expected number of MLGs based on rarefaction to a sample size of 50; Host‐specific, non‐singleton MLGs only observed in the given host; *N*, number of individuals with a complete genotype (no missing data); Shared, MLGs shared between tobacco and one other crop; Singletons, MLG observed only once.

The genetic differentiation among the 75 populations with at least 10 individuals genotyped, as measured by Weir and Cockerham's *F*_ST_, was 0.807 (95% CI: 0.662–0.865). An AMOVA was conducted on the data for the three most represented host crops: hemp, oilseed rape from France and tobacco. It revealed highly significant genetic differentiation among the different host crops and among populations within host crops, with the highest amount of genetic variation (approximately 70%) found among host crops (Table 3). Jost's *D* value was 0.480 (95% CI 0.477–0.482) for the 75 populations. Similar values of Jost's *D* were observed among populations sampled in hemp (*D* = 0.152; 95% CI 0.148–0.157) and in tobacco (*D* = 0.150; 95% CI 0.147–0.153), while significantly lower *D* values were observed among populations sampled in oilseed rape fields in France (*D* = 0.065; 95% CI 0.059–0.070). The Mantel's tests performed separately for each of the three host crops were all non‐significant, indicating that there was no isolation by distance among populations.

**TABLE 3:** Results of the AMOVA on microsatellite data for 64 populations collected on hemp (10 populations), oilseed rape in France (31 populations), and tobacco (32 populations).

Source of variation	*df*	Variance component	*F*‐statistics (95% CI)
Among host crops	2	5.17	0.710 (0.545–0.874)
Among population within host crops	68	1.32	0.625 (0.516–0.737)
Within populations	1294	0.948	0.884 (0.649–0.951)

### 3.3 Genetic clustering

**FIGURE 6:**
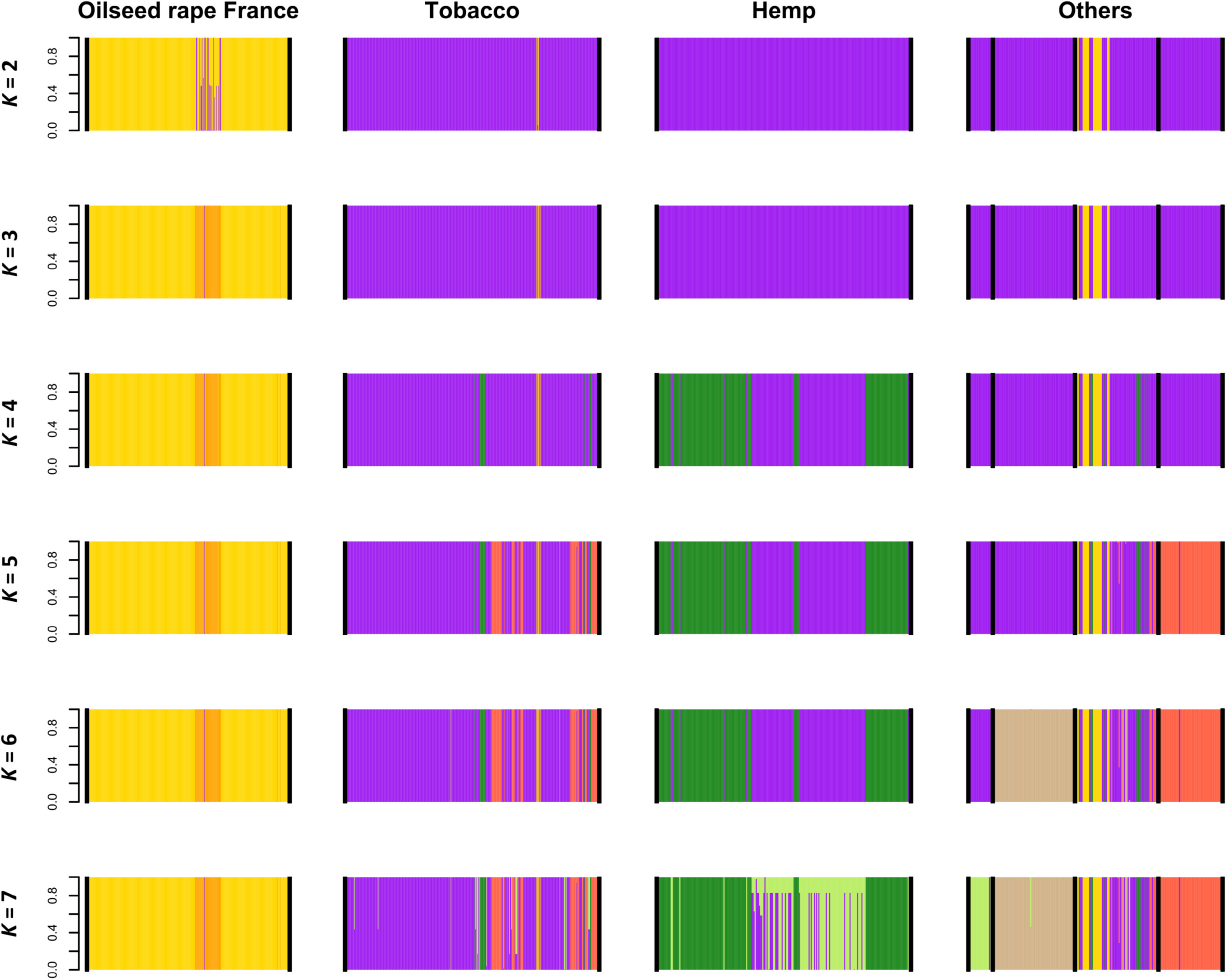
Results of a discriminant analysis of principal components (DAPC) conducted on microsatellite data considering from *K* = 2 to *K* = 7 genetic clusters. Each genotyped individual is represented by a vertical bar colored according to its membership coefficient for each of the *K* clusters. For each value of *K*, individuals are grouped according to the host crop from which they were sampled (indicated at the top of the barplots). The group indicated as “Others” includes the following host crops, from left to right: celery, lentil, oilseed rape from Greece and tomato.

**FIGURE A1:**
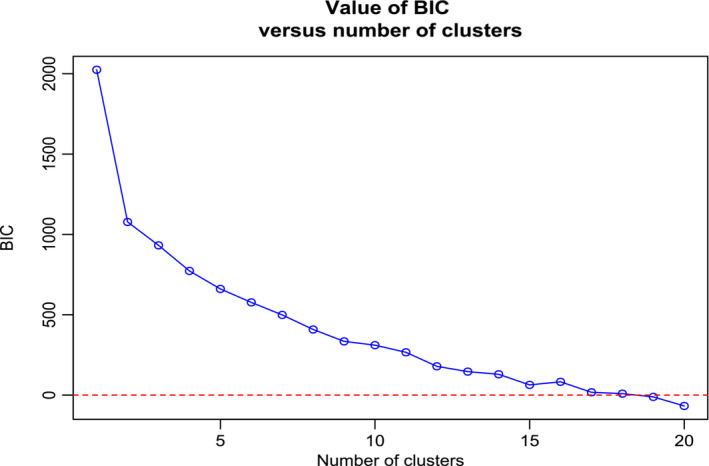
Value of the Bayesian Information Criterion (BIC) as a function of the number of genetic clusters considered in the DAPC analyses among *Phelipanche ramosa* accessions genotyped at 10 microsatellites.

**FIGURE A2:**
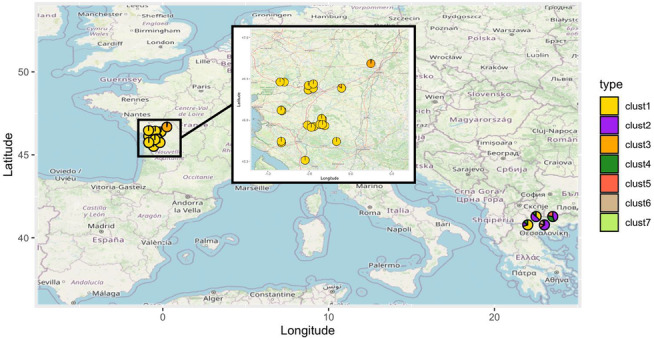
Geographical map showing the distribution of the genetic clusters identified by DAPC at *K* = 7 in the *Phelipanche ramosa* populations sampled from oilseed rape fields.

**FIGURE A3:**
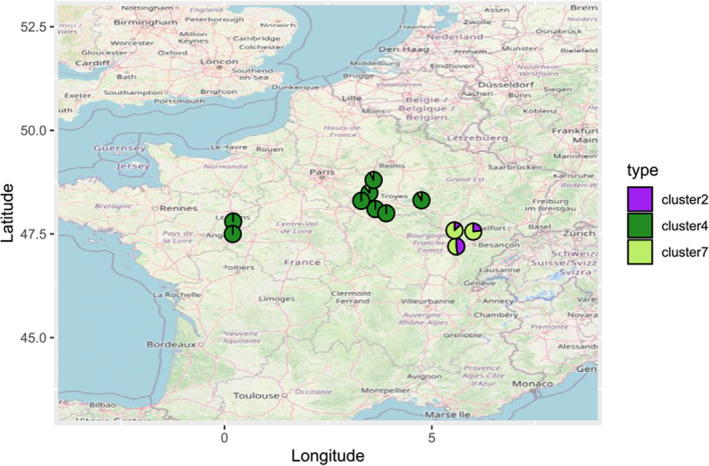
Geographical map showing the distribution of the genetic clusters identified by DAPC at *K* = 7 in the *Phelipanche ramosa* populations sampled from hemp fields.

**FIGURE A4:**
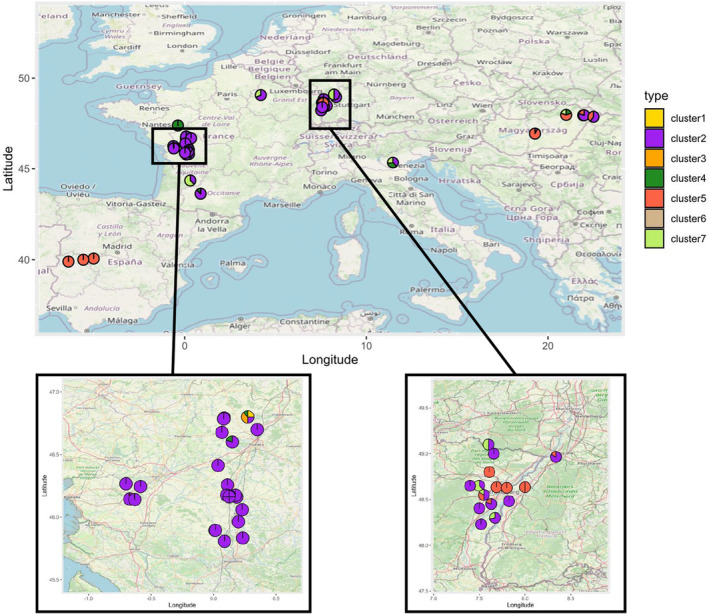
Geographical map showing the distribution of the genetic clusters identified by DAPC at *K* = 7 in the *Phelipanche ramosa* populations sampled from tobacco fields.

The k‐means clustering algorithm implemented in DAPC could not identify an optimal number of genetic clusters, as the Bayesian Information Criterion decreased continuously with the number of clusters (Appendix A: Figure A1). We retained the first seven clusters identified, as they showed association with the host crops (Figure 6). At *K* = 2, cluster 1 (yellow color on Figure 6) grouped all the individuals sampled in oilseed rape fields from France, a few individuals from one tobacco field and some individuals from oilseed rape fields in Greece. At *K* = 3 a new genetic cluster (cluster 3) was associated with one oilseed rape field from France, phera41, which had also an intermediate position in the phylogeny (Figure 4). Examination of genotypes showed that this cluster resulted from admixture between clusters 1 and 2. As *K* increased above 3, clusters associated with oilseed rape from France remained unchanged while new clusters were successively defined among the other crops. Most of the individuals collected on tobacco were assigned to cluster 2 (purple color on Figure 6). Cluster 4 (dark green color on Figure 6) was found in a majority of the individuals sampled on hemp. Cluster 5 (red color on Figure 6) was found in all individuals sampled on tomato and some individuals sampled on tobacco and oilseed rape in Greece. Cluster 6 (brown color on Figure 6) was found in all individuals sampled on lentil, as well as a few individuals from oilseed rape fields in Greece. Cluster 7 (light green color on Figure 6) was found in individuals sampled in a celery field and some hemp and tobacco fields. The geographical distribution of the clusters among oilseed rape, hemp, and tobacco fields is shown in Appendix A: Figures A2, A3, A4.

### 3.4 Migration and admixture

In 70% of the populations (76 among 109), only one genetic cluster was observed. In the other populations, different patterns could be observed: (i) the presence of likely migrants, that is, genotypes assigned with a membership probability higher than 0.9 to a genetic cluster different from the main cluster in their population, (ii) a mix of genotypes assigned (with a threshold higher than 0.9) to different clusters, and (iii), the presence of admixed genotypes, each assigned to two or more clusters with thresholds lower than 0.9. The second pattern was observed in all four oilseed rape fields sampled in Greece, where 42% of the individuals were assigned to cluster 2, 26.5% to cluster 1, 15.6% to cluster 4, 9.4% to cluster 5, 4.7% to cluster 6 and 1.5% to cluster 7. This suggested that these fields were recently colonized from multiple origins. The third pattern (admixture) was rare, as only 2.9% admixed individuals were observed overall. Except for one individual admixed between the clusters 6 (associated with lentil) and 7 (associated with hemp), all observed admixture events involved the genetic clusters 2 (associated with tobacco) and 7 (associated with hemp) and were observed in 3 hemp fields and 6 tobacco fields.

GENECLASS identified first‐generation migrants based on a priori defined populations (the agricultural fields), without considering the genetic clusters identified by DAPC. Overall, 85 migrants were identified in 52 populations (Table 4), and the average migration rate per population was 9%. Migrants assigned to genetic cluster 1 (associated with oilseed rape) were only observed within oilseed rape fields. Migrants assigned to cluster 2 (associated with tobacco) were observed within tobacco fields, hemp fields, tomato fields, one celery field, and one oilseed rape field. Migrants assigned to genetic clusters 4 and 7 (associated with hemp) were observed within hemp fields and tobacco fields. Migrants assigned to cluster 5 (associated with tomato) were observed within tobacco fields and tomato fields. Migrants assigned to genetic cluster 6 (associated with lentil) were only observed within lentil fields.

**TABLE 4:** First‐generation migrants identified by GENECLASS within populations of *Phelipanche ramosa*.

Host crop of the home population	Host crop of the source population	Number of populations with migrants	Average migration rate	Home genetic clusters	Migrant genetic clusters
Celery	Tobacco	1	0.053	7	2
Hemp	Hemp	7	0.076	4, 7, 2	4, 7, 2
	Tobacco	4	0.057	4, 7, 2	4, 2
Lentil	Tobacco	2	0.125	6	6
Oilseed rape (France)	Oilseed rape (France)	13	0.124	1	1
	Tobacco	3	0.093	1, 3	1, 2
Tobacco	Tobacco	18	0.171	2, 5, 7	2, 5, 7
	Hemp	1	0.222	4	4
Tomato	Tobacco	2	0.126	5	5, 2
	Tomato	1	0.042	5	2

### 3.5 Historical evolutionary scenarios

**FIGURE 7:**
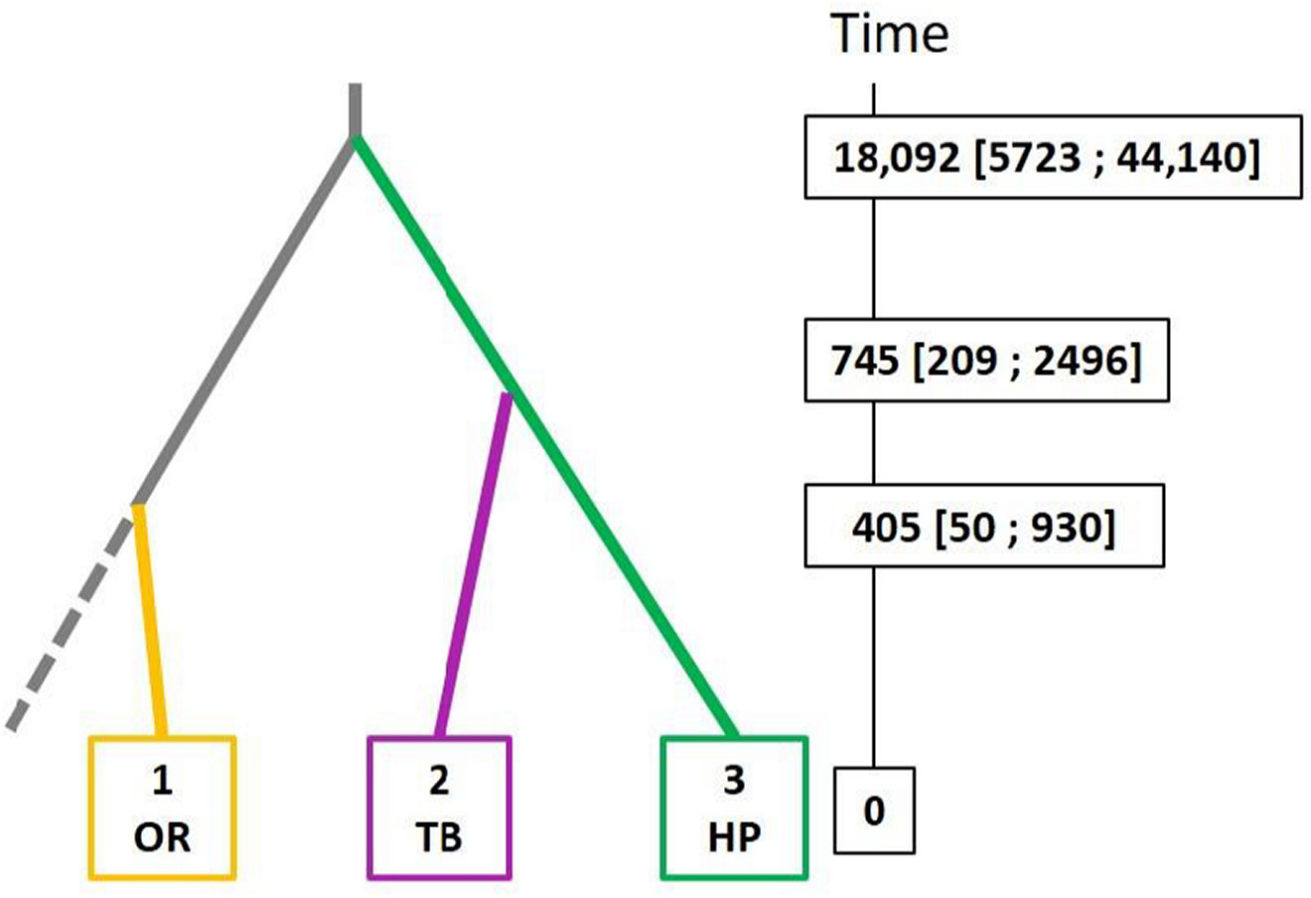
The most likely evolutionary scenario of divergence as inferred from Approximate Bayesian Computation for the 3 main, non‐admixed genetic clusters identified by DAPC analyses (OR: Oilseed rape, TB: Tobacco, HP: Hemp). Median values and 95% confidence intervals for the estimated times of divergence (in number of generations) are shown.

**FIGURE A5:**
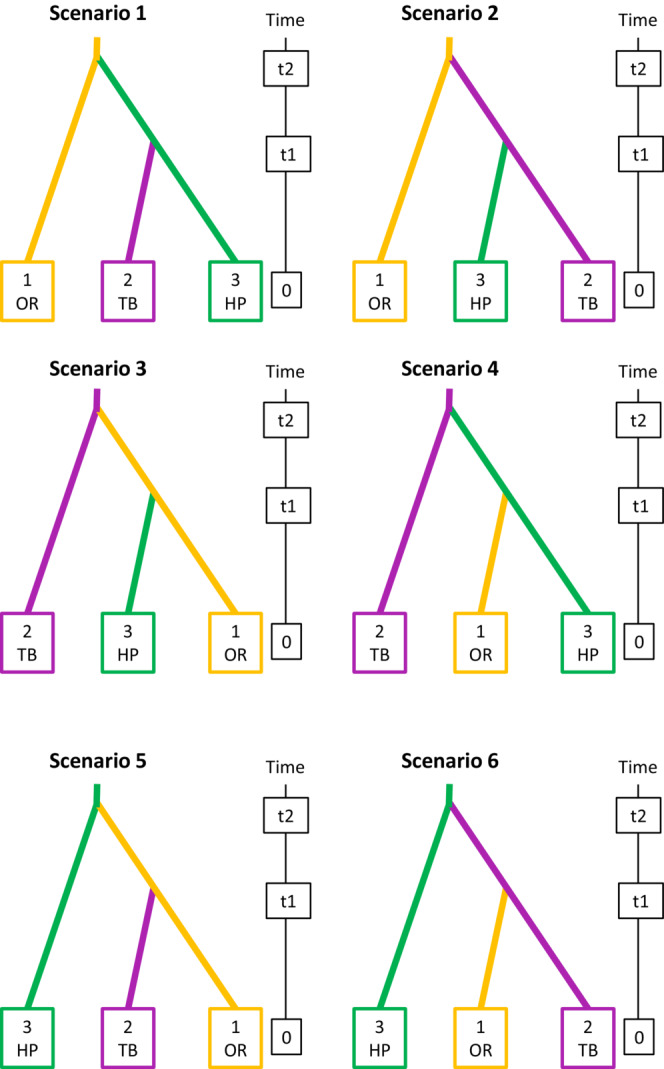
The six possible evolutionary scenarios for the three main genetic clusters identified by DAPC and associated with the three main host crops (OR: oilseed rape, TB: tobacco, HP: hemp).

**FIGURE A6:**
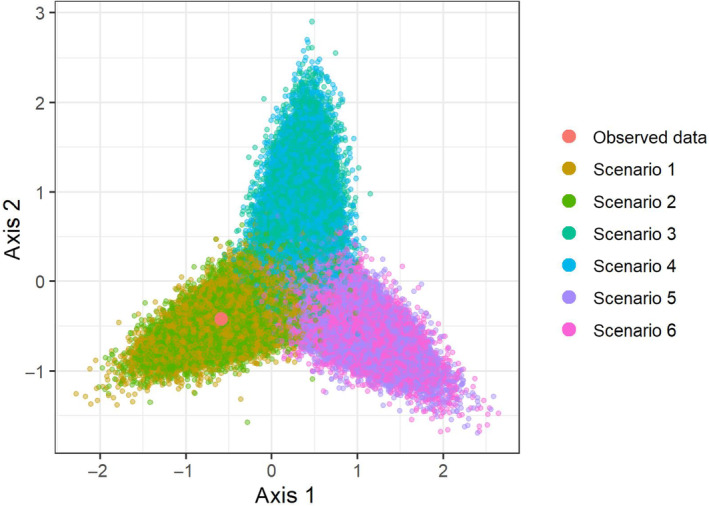
Linear discriminant analysis of the simulated training datasets obtained for each of the six scenarios considered. The red dot indicates the location of the observed dataset.

**FIGURE A7:**
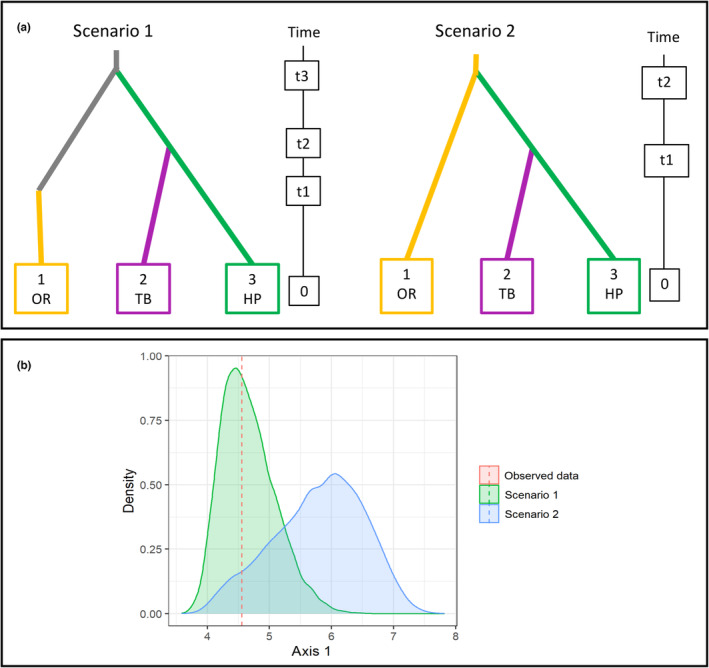
Comparison of two scenarios for the best‐supported divergence model: (a) scenario 1 includes a ghost, unsampled population as the ancestor of the OR genetic cluster, while scenario 2 does not, (b) Projection on an LDA axis of the training data sets generated for each scenario. The location of the observed data set is indicated by a vertical dashed line.

As a first step, we evaluated all the six possible divergence scenarios (Appendix B: Figure A5) involving the three main genetic clusters detected by DAPC at *K* = 4: the cluster associated with oilseed rape from France (hereafter OR), the cluster associated with hemp (hereafter HP), and the cluster associated with tobacco and other crops (hereafter TB; note that at *K* = 7, this cluster is further subdivided and includes the clusters 5, 6 and 7). We did not include genetic cluster 3, only observed in one oilseed rape population from France (phera41), as it seemed to result from an admixture between cluster 1 and cluster2 (Figure 6). The two scenarios that best matched the observed data (Appendix B: Figure A6) and collected the highest classification votes from Random Forest computations were those with the most ancient divergence event between the ancestor of OR and the ancestor of TB (45.2% of votes on average across 10 replicates) or between the ancestor of OR and the ancestor of HP (52.9% of votes on average across ten replicates). In each of the ten replicated Random Forest analyses, the scenario with the highest number of votes was the latter. The mean posterior probability for this scenario was 0.565. Demographic and historical parameters were estimated for this best scenario, slightly modified by introducing a ghost, unsampled population from which population OR derived. The scenario with a ghost population was consistently preferred over the initial scenario without the ghost population across a set of ten replicate Random Forest analyses (Appendix B: Figure A7). Median values and 95% confidence intervals for times of divergence in this scenario are shown on Figure 7 (values of local and global accuracy indices are reported in Appendix B: Table A1). Despite the large confidence intervals around estimated times of divergence, results suggested that the oilseed rape‐associated genetic cluster originated a few hundreds of generations ago (estimated median: 405) from an unknown, ancient lineage. The divergence between the TB and HP clusters occurred earlier (estimated median: 745). The divergence between the ancestor of the OR cluster and the common ancestor of the TB and HP cluster was ancient, about 18,000 generations ago.

## 4 DISCUSSION

The success of a parasite requires a combination of traits that maximize adaptation to the biology and physiology of its host. Parasites may specialize on a single host or be able to parasitize multiple host species. In the latter case, the divergent selection exerted by the different hosts may result in some genetic divergence among sympatric subpopulations of the parasite, a phenomenon called host‐associated genetic differentiation. Host‐associated genetic differentiation has been well documented, for example, in parasitic arthropods (Harrison et al., 2021) and especially in phytophagous insects including agricultural pests (e.g., aphids: Peccoud et al., 2009). Parasitic plants have been comparatively much less studied. Among parasitic plants of agronomic importance, host‐associated genetic differentiation has been reported in *Striga hermontica* (Unachukwu et al., 2017); *Orobanche crenata* (Bendaoud et al., 2022) and *Orobanche foetida* (Boukteb et al., 2021). Due to its broad host range and agronomic importance, the genetic variation of *P*. *ramosa* has been already well characterized (Benharrat et al., 2005; Brault et al., 2007; Le Corre et al., 2014; Stojanova et al., 2019). Here, we expanded on previous studies to characterize host‐associated differentiation more in detail and understand the origin of a recent host shift.

### 4.1 Strong host‐associated genetic differentiation in *Phelipanche ramosa*

Our population genetic analyses demonstrated that the genetic variation in *P*. *ramosa* is mainly structured by host plant species. Our results confirm and extend those of previous studies. As already described (Benharrat et al., 2005; Brault et al., 2007; Le Corre et al., 2014; Stojanova et al., 2019), populations of *P*. *ramosa* that infest oilseed rape fields in western France belong to a highly differentiated genetic group (cluster 1 in our analyses). In our study, this genetic group was not observed in any other host crop or geographical region, except in some populations sampled from oilseed rape fields in northern Greece. Cluster 1 is very likely to be identical to group 1 previously described by Stojanova et al. (2019). In their study, this group was observed mainly in oilseed rape fields in western France, with a few rare occurrences in tobacco, melon, and sunflower, also in western France. Two other genetic groups have been described in previous studies and have been associated mainly with populations parasitizing hemp or tobacco. Stojanova et al. (2019) observed the first group (group 2a in their study) in populations sampled from hemp, tobacco, tomato, and oilseed rape fields in eastern France. They observed the second group (group 2b in their study) in populations sampled from tobacco, hemp, celery, and tomato. In the present study we have extended the number of genetic clusters examined up to seven and showed that the additional clusters obtained were all associated with specific host crops. Comparison with the results from Stojanova et al. (2019) suggests that their group 2a is identical to our cluster 4 (associated mainly with hemp), while their group 2b encompasses our clusters 2 (associated mainly with tobacco), 5 (associated with tomato and tobacco) and 7 (associated with hemp, celery, and tobacco). In addition, we observed a genetic cluster (cluster 6) that was associated with lentil fields in Greece. No previous study had analyzed the genetic composition of *P*. *ramosa* populations sampled from a legume crop so that it seems that this genetic group had not been detected before. The analysis of a larger number of populations is necessary, however, to determine whether this genetic group is associated with lentil only, with several host species belonging to the *Fabaceae* family or to a more diverse range of host species. *P*. *ramosa* has been reported to be able to parasitize chickpea, clovers, groundnut, faba bean, lentil, and pea, but yield damages have been much less commonly reported in these crops than in oilseed rape, hemp, tobacco, or tomato (Parker, 2009, 2013). Compared to previous studies, we have thus identified three additional genetic clusters associated with different host crops. The host preference of each of these additional clusters will need to be investigated using phenotyping assays, which was not possible in the present study.

### 4.2 Migration among populations is constrained by host preference

The allelic diversity and genotypic diversity among plants sampled within a given cultivated field was low, with observed values very similar to those reported by Stojanova et al. (2019). This is consistent with the predominantly selfing mating system of the species (Román et al., 2003) and is also expected if new infestations are mainly caused by single colonization events. The migration rate as estimated from the identification of first‐generation migrants was surprisingly high (9%) in comparison to the observed value of genetic differentiation among populations (*F*_ST_ = 0.81). Host specialization may filter out genotypes not adapted to the local host crop. Indeed, migration events in which incoming seeds were assigned to a different genetic cluster than the receiving population were comparatively much less frequent than migration events in which the same genetic cluster was involved. As a result, most migrants are more genetically similar to individuals already present in the recipient populations than would be expected if migration was random.

In western France, where both oilseed rape and tobacco are grown within the same area, first‐generation migration events provide indirect information about the host preference of the genetic clusters. Migrant assigned to cluster 1 were never observed in tobacco. This confirms that genetic cluster 1 is tightly associated with oilseed rape. This strong host specificity may be due to an adaptive evolution toward a longer life cycle matching the duration of the winter crop cycle of oilseed rape but not the shorter, summer cycle of tobacco, hemp, or tomato (Stojanova et al., 2019). Conversely, a few migrants assigned to cluster 2 (associated with tobacco) were observed in some oilseed rape fields. This is consistent with previous co‐culture experiments where *P*. *ramosa* collected from a tobacco field was shown to be able to germinate and grow on various species of *Brassicaceae* (Gibot‐Leclerc et al., 2015).

In contrast to cluster 1, cluster 2 is the most versatile genetic group in terms of adaptation to the host species, as genotypes assigned to this cluster were observed in all crops except lentil and celery (for which only 3 and 1 fields, respectively, were included in our study). Both cluster 5 (associated with tomato) and cluster 7 (associated with hemp) were found within tobacco fields, which is consistent with the fact that they are genetic subgroups within cluster 2. The close genetic proximity between clusters 2, 5, and 7 was attested by the observation of admixed individuals. In addition, shared multilocus genotypes were observed and first‐generation migrants were detected between populations sampled on tobacco, hemp, and tomato. These results are consistent with co‐culture experiments by Stojanova et al. (2019), where their cluster 2b, which probably includes our clusters 2, 5, and 7, was found to have almost equal growth success on tobacco, hemp, and tomato.

### 4.3 Long‐distance dispersal and the origin of populations infesting oilseed rape in Greece

Mantel tests gave no indication of isolation by distance among fields cultivated with the same crop. On the other hand, a same multilocus genotype could be observed in very distant populations, for example in tomato fields from Greece and tobacco fields from Spain, and several MLGs were shared between tobacco fields from eastern France, western France, and Hungary. Long‐distance dispersal is likely very important in distributing genetic variation among *P*. *ramosa* populations at the European scale, maybe due to human transportation of crop seeds and harvests. Populations sampled in oilseed rape fields in Greece had an outstanding pattern of genetic variation, as they contained a mix of genotypes assigned to all genetic clusters. This suggested some migration events from other fields cultivated with different crops, and/or the emergence of a diversity of genotypes from the seedbank, especially if a variety of host crops had been cultivated locally in the past. These Greek oilseed rape fields were the only locations outside of western France where we observed the oilseed rape genetic cluster, so that long‐distance dispersal of *P*. *ramosa* seeds between western France and northern Greece is a possibility. More details on the past agronomic history of the fields would be needed to better understand how they were colonized by *P*. *ramosa*. Likewise, some future temporal monitoring and resampling of the populations would allow the evolution of their genetic composition and host preference to be tracked.

### 4.4 The evolutionary history of *P*. *ramosa* and origin of populations infesting oilseed rape in western France

Oilseed rape is a recently developed crop. It did not become a major crop in France until the 1980s, when double‐zero varieties (low in glucosinolates and erucic acid), were introduced. The first infestations of *P*. *ramosa* in this crop were observed as early as the beginning of the 1990s and were thus considered to be the result of a recent and rapid host shift (Stojanova et al., 2019). However, our phylogenetic analyses showed that *P*. *ramosa* accessions sampled from oilseed rape were grouped in the most basal lineage in the evolutionary tree of the species. In addition, evolutionary scenario testing using Approximate Bayesian Computation ruled out the hypothesis that the genetic cluster associated with oilseed rape as a host was derived from either of the other two main genetic clusters. On the contrary, the best‐supported scenario indicated that an unknown lineage diverged early from the ancestor of both the tobacco‐associated and hemp‐associated lineages, accumulated large genetic differences and was recently the source of a genetic population capable of parasitizing oilseed rape. It is intriguing that this ghost lineage or other recent populations derived from it were not identified in our study nor in any previous studies. Some hypotheses can be proposed. First, the ghost populations may be located in an as yet unstudied geographical area. In both our study and the previous large‐scale study by Stojanova et al. (2019), very few populations from around the Mediterranean basin were analyzed. In particular, it would be necessary to analyze more populations from south‐eastern Europe, Turkey, and the Middle East as well as North Africa, to obtain a more comprehensive picture of the genetic variation in *P*. *ramosa*, as the species is considered to be native to all these areas (Parker, 2013). On the other hand, as *P*. *ramosa* has an extremely wide host range that includes many wild or weedy plants, it is possible that the ghost populations are located mainly outside cultivated fields or do not parasitize crops, and for this reason were not sampled. The possibility of host shifts from a wild or weedy host to a cultivated host may have important consequences for the management of the species and deserves to be investigated. A first step to test this hypothesis would be to investigate the occurrence and genetic identity of *P*. *ramosa* parasitizing wild or weedy plant species from the *Brassicaceae* family.

## 5 CONCLUSION

Our study confirms that the agronomically important parasitic plant *P*. *ramosa* is a generalist species whose genetic diversity is mainly structured by host preference. A complex pattern of host‐associated genetic differentiation is observed, so that classification into discrete pathovars seems impossible. A highly specialized genetic group infesting oilseed rape coexists with more generalist, non‐exclusive host‐associated genetic groups and subgroups with an overlapping continuum of host ranges. Most importantly, our study suggests that the recent shift to oilseed rape as a new host originated from an ancient, unknown source population that may have been pre‐adapted. The description of genetic variation in the species is, therefore, likely to be far from complete. Our results support the need to further investigate the genetic composition of *P*. *ramosa* populations from under‐sampled geographic regions and host plants, especially wild and weedy hosts, as this may lead to the identification of reservoirs for future adaptations and host shifts to new crops.

## AUTHOR CONTRIBUTIONS

**Valérie Le Corre:** Conceptualization (equal); data curation (equal); formal analysis (lead); investigation (equal); methodology (equal); writing – original draft (lead). **Carole Reibel:** Data curation (equal); investigation (equal); methodology (equal); writing – review and editing (supporting). **Vaya Kati:** Data curation (equal); investigation (equal); methodology (supporting); writing – review and editing (equal). **Stéphanie Gibot‐Leclerc:** Conceptualization (equal); data curation (equal); funding acquisition (lead); investigation (equal); methodology (supporting); writing – review and editing (equal).

## CONFLICT OF INTEREST STATEMENT

The authors declare no competing interest.

## Data Availability

Details of the plant material, microsatellite genotypes, and DNA sequences are available at Zenodo: https://doi.org/10.5281/zenodo.8073917. The data that support the findings of this study are available from the corresponding author upon reasonable request.

## APPENDIX A

### A.1

**Genetic clustering analysis with DAPC**

## APPENDIX B

### B.1

Comparison of evolutionary scenarios with Approximate Bayesian Computations using DIYABC Random Forest v1.0

**TABLE A1:** Estimated historical and demographic parameters under the best scenario of divergence (scenario 1 on Figure A7).

Parameter	Median	95% CI	Global NMAE	Local NMAE	90% coverage
*t*1	405 (14.34)	50–930 (4.35) (7.95)	1.603 (0.04)	1.762 (0.24)	0.905 (0.002)
*t*2	745 (29.12)	209–2496 (10.32) (111.74)	0.425 (0.01)	0.685 (0.09)	0.912 (0.002)
*t*3	18,092 (765.71)	5723–44,140 (158.01) (748.68)	0.401 (0.002)	0.704 (0.09)	0.910 (0.003)
*N*1 (OR)	743 (13.06)	286–974 (11.38) (2.92)	0.374 (0.003)	0.285 (0.03)	0.924 (0.003)
*N*2 (TB)	796 (8.08)	396–980 (9.50) (3.70)	0.327 (0.01)	0.296 (0.22)	0.922 (0.003)
*N*3 (HP)	479 (11.88)	199–908 (7.57) (12.36)	0.318 (0.004)	0.359 (0.04)	0.920 (0.003)

*Note*: Means and standard deviations (within brackets) over ten replicates of the analyses are given for each metric.
